# Supplementary material for: Assay validation and determination of in vitro binding of mefloquine to plasma proteins from clinically normal and FIP-affected cats
Source: PLoS One. 2020 Aug 5;15(8):e0236754. doi: 10.1371/journal.pone.0236754 (PMC7406051; doi:10.1371/journal.pone.0236754)
Supplement: Attachment — Submitted filename: PONE-D-20-08739docx.docx [file pone.0236754.s001.docx]

PONE-D-20-08739

The manuscript entitled “Assay validation and determination of in vitro binding of mefloquine to plasma proteins from clinically normal and FIP-affected cats” by Izes et al., describes the development of quantitative assay for mefloquine hydrochloride by HPLC. Verapamil hydrochloride was used as the internal standard. The validated assay was then applied to quantitate amount of plasma protein bound mefloquine hydrochloride from normal and FIPV infected cats. The plasma samples were from clinically normal and FIP-affected cats. The researchers pooled 5 samples of each clinical type of plasmas and differentiated them by using albumin:globulin (A:G) ratio. Mefloquine was spiked into the plasma samples and plasma protein bound mefloquine hydrochloride was quantitated by rapid equilibrium dialysis (RED) method. A:G ratio is a non-specific and rough method to diagnose FIP cats based on the fact that immunoglobulin was exacerbated in FIP-affected cats. The authors should use RT-PCR to detect the viral nucleic acid in the plasma to confirm the diagnosis. Majority of contents in the manuscript are related to the assay development and validation which is not interesting as HPLC is not new technology. The authors did not compare this assay with other methods and show how attractive it is. In addition, not much assay application was described.
