# Supplementary material for: Assay validation and determination of in vitro binding of mefloquine to plasma proteins from clinically normal and FIP-affected cats
Source: PLoS One. 2020 Aug 5;15(8):e0236754. doi: 10.1371/journal.pone.0236754 (PMC7406051; doi:10.1371/journal.pone.0236754)
Supplement: Attachment — Submitted filename: Response to reviewers PONE-D-20-8739 - JN_AI.docx [file pone.0236754.s002.docx]

PONE-D-20-08739
Assay validation and determination of in vitro binding of mefloquine to plasma proteins from clinically normal and FIP-affected cats
PLOS ONE

Dear Editor

Thanks to you and the Reviewers for taking the time to review the manuscript and your comments.

The authors respond to the comments:

**Journal requirements:**

**When submitting your revision, we need you to address these additional requirements.**

**1. Please ensure that your manuscript meets PLOS ONE's style requirements, including those for file naming. The PLOS ONE style templates can be found at**

[https://journals.plos.org/plosone/s/file?id=wjVg/PLOSOne_formatting_sample_main_body.pdf](https://protect-au.mimecast.com/s/Z5wwC1WLPxc8qQm5up40Em?domain=journals.plos.org) and

***Response:*** *The manuscript has been revised to comply with PLOS one formatting*

[https://journals.plos.org/plosone/s/?id=ba62/PLOSOne_formatting_sample_title_authors_affiliations.pdf](https://protect-au.mimecast.com/s/zuVUC2xMQzi78DGOt2tkU_?domain=journals.plos.org) file

***Response:*** *The authors have revised their affiliations*

**2. In your methods section please state whether informed consent of cat owners was obtained, and if so, if consent was written or verbal.**

 Accordingly this aspect has been revised to:

***Response:*** *‘With owner verbal or written consent, blood was collected from both clinically normal and FIP-affected cats. The use of the residual plasma from this patient for this study was approved by The University of Sydney Animal Ethics Committee (protocol: 2016/1027).’*

See lines 143 to 145.

Reviewers' comments:


**5. Review Comments to the Author**

**Reviewer #1: The manuscript entitled “Assay validation and determination of in vitro binding of mefloquine to plasma proteins from clinically normal and FIP-affected cats” by Izes et al., describes the development of quantitative assay for mefloquine hydrochloride by HPLC. Verapamil hydrochloride was used as the internal standard. The validated assay was then applied to quantitate amount of plasma protein bound mefloquine hydrochloride from normal and FIPV infected cats. The plasma samples were from clinically normal and FIP-affected cats. The researchers pooled 5 samples of each clinical type of plasmas and differentiated them by using albumin:globulin (A:G) ratio. Mefloquine was spiked into the plasma samples and plasma protein bound mefloquine hydrochloride was quantitated by rapid equilibrium dialysis (RED) method. A:G ratio is a non-specific and rough method to diagnose FIP cats based on the fact that immunoglobulin was exacerbated in FIP-affected cats. The authors should use RT-PCR to detect the viral nucleic acid in the plasma to confirm the diagnosis. Majority of contents in the manuscript are related to the assay development and validation which is not interesting as HPLC is not new technology. The authors did not compare this assay with other methods and show how attractive it is. In addition, not much assay application was described**.

*Re the comment that the authors should use RT-PCR to detect viral nucleic acid in the plasma:

***Response:*** *RT-PCR to detect viral nucleic acid of feline coronavirus in the blood is not an agreed method of diagnosis for FIP and is not recommended by any of the veterinary diagnostic laboratories globally due to the low sensitivity and low specificity. Cats in the primary phase of simple feline enteric coronavirus infection will have a transient viraemia and none of the current PCR techniques differentiate this (Porter et al. Veterinary Research 2014, 45:49). We used immunohistochemistry and direct immunofluorescence to confirm the diagnosis of FIP. Four of the cats were diagnosed with FIP by immunohistochemistry and the other five by both immunohistochemistry and immunofluorescence assay (IFA) on infusions. Additionally, other biochemical tests were performed for each cat such as quantification of total plasma proteins, albumin and globulins (including globulin subfractions) as reported in Worthing et al., 2012 and has been included in the manuscript, see lines 156 - 158.*

*Re the comment that the majority of contents in the manuscript are related to assay development and validation which is not interesting as HPLC is not new technology.

***Response:*** *The authors cannot agree with this statement. Our laboratory undertakes drug development and validation. Both of these processes can take a long time and this research develops a protocol that many other researchers can use. This research describes a sensitive, validated HPLC method to detect mefloquine in feline plasma. That in itself is novel and should be of interest to those involved in feline research and save such researchers the time in developing such an assay.*

Re the comment that the authors did not compare this assay with other methods and show how attractive it is. In addition, not much assay application was described.

***Response:*** *The majority of publications that detect and quantify mefloquine use liquid chromatography*. *Liquid chromatography demonstrates good sensitivity (i.e. it can detect low concentrations of mefloquine), is highly specific and the machinery is affordable for veterinary diagnostic laboratories.*

*This information has been added to the manuscript see lines 276 – 279.*

Re the comment that not much assay application was described.

***Response:*** *This is a surprising comment as this assay was used to detect the mefloquine concentrations in the plasma protein binding study.*

**Reviewer #2:
Reviewer’s comments:
This manuscript is described that assay validation and determination of binding of mefloquine to plasma proteins. The manuscript is well written as a whole. However, I have a minor question on this manuscript. Therefore, please ask the question.

Minor comments;
The authors used mefloquine and verapamil for this assay but I wonder why you used verapamil. You did not show verapamil data in the Figs. and Tables in the manuscript. Therefore, you should explain the research object to use verapamil and show any data on verapamil studies.**

***Response:*** *Verapamil was used as the internal standard as mentioned in line 77. This means the same concentration of verapamil was added to every sample. If the concentration of verapamil changes during the assay it indicates that there is a system problem. Either 12.5 µg/mL of the verapamil was added to the validation study or 5.0 µg/mL of the verapamil was added to each sample for the plasma protein binding study. Therefore a known concentration of verapamil was added to for each sample.*

*Verapamil was used in this study as it was used the internal standard used by Gbotosho et al., (2012* ([http://www.malariajournal.com/content/11/1/59](https://protect-au.mimecast.com/s/Peh9CBNqjlCJ9om1Fzru76?domain=malariajournal.com)).

**Reviewer #3:**

**In this paper, the authors propose a method for measuring mefloquine binding to feline plasmatic proteins, which is fundamental to assess the in vivo efficacy of this drug in cats with feline infectious peritonitis (FIP). Although the study presents some limitations, mainly related to the in-vivo adaptation of in-vitro model, it deserves publication after few issues are addressed.


Introduction. A brief introduction of the virus, its characteristics and the caused disease (FIP) would be beneficial to the general audience of the journal. See Decaro and Lorusso (Vet. Microbiol. 2020,** [**https://doi.org/10.1016/j.vetmic.2020.108693**](https://protect-au.mimecast.com/s/FM8LC4QOPEirlwW3UMSXZU?domain=doi.org)**) for an overview.
Blood collection. The authors should provide details on how many FIP cases were diagnoses using immunofluorescence assay (IFA) on effusions and how many using immunohistochemistry, which is the gold standard for FIP diagnosis. The authors should consider that IFA is not the gold standard and that an IFA-based diagnosis should be supported by additional evidence according to the European Advisory Board of Cat Disease recommendations, including Rivalta’s test, total proteins, albumin/globulin ratio, total leukocyte counts and identity of cells (Lorusso et al., Res Vet Sci. 2019 Aug;125:421-424).
Discussion. A more detailed discussion about the comparative analysis with COVID-19 therapy should be presented.**

*Re the comment that the authors should provide details on how many FIP cases were diagnoses using immunofluorescence assay (IFA) on effusions and how many using immunohistochemistry, which is the gold standard for FIP diagnosis.

***Response:*** *The diagnosis of FIP was confirmed by both immunohistochemistry and direct immunofluorescence on five cats and by immunohistochemistry alone on four cats. Additionally, other biochemical tests were performed such as quantification of total plasma proteins, albumin and globulins (including globulin subfractions were determined for each cat) as reported in Worthing et al., 2012. However, the relevant issue was to determine the proportion of drug binding to feline plasma with albumin/globulin ratio within the normal range verses feline plasma with a lower albumin / globulin ratio. This information about plasma proteins has been added to lines 156 to 158.*

Re the comment for a brief introduction of the virus, its characteristics and disease would be beneficial to the general audience of the journal. The audience of this journal is not feline orientated, however the reader has been directed to relevant reviews as follows

***Response: Accordingly, the following has been included:***

*Reviews describing the virus responsible for FIP as well as issues with diagnostics and therapeutics are available [1, 2], providing an explanation as to why re-purposing drugs used in human medicine has been largely unsuccessful in treating FIP infected cats [1].*

*See lines 44 – 47.*

*1.	Pedersen NC. An update on feline infectious peritonitis: diagnostics and therapeutics. Vet J. 2014;201:133-41.*

*2.	Pedersen NC. A review of feline infectious peritonitis virus infection: 1963–2008. J Feline Med Surg. 2009;11:225-58.*

Re comment - Discussion. A more detailed discussion about the comparative analysis with COVID-19 therapy should be presented.

***Response:*** *The authors’ appreciate the Reviewer’s guidance but we do not think this manuscript directly addresses COVID-19 issues and thus incorporating this suggestion will confuse readers.*
